# Electrochemical [^11^C]CO_2_ to [^11^C]CO conversion for PET imaging†
†Electronic supplementary information (ESI) available. See DOI: 10.1039/c7cc00319f



**DOI:** 10.1039/c7cc00319f

**Published:** 2017-02-24

**Authors:** David A. Anders, Salvatore Bongarzone, Robin Fortt, Antony D. Gee, Nicholas J. Long

**Affiliations:** a Department of Chemistry, Imperial College London, London SW7 2AZ, UK. Email: n.long@imperial.ac.uk; b Division of Imaging Sciences and Biomedical Engineering, King's College London, 4th Floor Lambeth Wing, London SE1 7EH, UK

## Abstract

The development of a novel electrochemical methodology to generate carbon-11 carbon monoxide ([^11^C]CO) from cyclotron-produced carbon-11 carbon dioxide ([^11^C]CO_2_) using Ni(cyclam) and Zn(cyclen) complexes is described. This methodology allows up to 10% yields of [^11^C]CO from [^11^C]CO_2_. Produced [^11^C]CO was subsequently converted to [^11^C]*N*-benzylbenzamide under mild conditions with a radiochemical purity (RCP) of >98%.

## Abstract 2

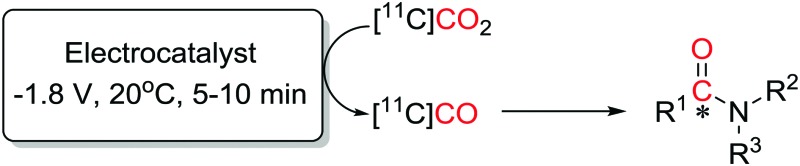
Development of a novel electrochemical radiochemistry methodology *i.e.* reduction of [^11^C]CO_2_ to [^11^C]CO at room temperature and pressure using metal cyclam complexes.

## 

Electrochemical reduction of CO_2_ to CO has long been considered an important issue for tackling environmental sustainability. The challenge lies in converting the thermodynamically stable CO_2_ molecule into more energetic compounds. The largest thermodynamic barrier is the first electron addition to convert the linear CO_2_ molecule to a bent anion radical (COO˙) at *E =* –1.90 V.^1^ So far, 2nd and 3rd row transition metal elements have dominated this area although only Au and Ag generate CO with Faradaic efficiencies (FE) above 80% and maintain high current densities.^2,3^ Their activities have been boosted by nanostructuring techniques controlling the surface morphology.^4^ Other cheaper metals such as Sn, Sb, Pb and Bi have been used to convert CO_2_ to CO with high current efficiencies using ionic liquids to stabilise the COO˙ intermediate.^5^ Ionic liquids have been increasingly studied for their role in lowering thermodynamic barriers in CO_2_ reduction and so too have group 1 cations such as K^+^ and Cs^+^.^6^

Homogenous electrocatalysts for CO_2_ reduction, such as cathode materials, have previously been dominated by metals such as Pd, Ru, Rh and Re.^7^ Over the last few decades, many complexes of the first row transition metal elements such as Fe, Co, Cr, Cu, Mn and Ni have been used as electrocatalysts. Most notably metal cyclams (metal = Ni and Co),^8^ metalloporphyrins (Fe, Co and Ni),^9^ metal polypyridines (Cr, Fe, Co and Ni), and metal phthalocyanines (Ni, Co, Mn, Fe and Cu).^10^ Most of these catalysts act as electron shuttles between the electrode and the CO_2_ molecule and so generally the metal is in a low oxidation state and the ligand stabilises the intermediates by some inner-sphere effect. Recently, several groups have also highlighted that the activity of these catalysts can be boosted by adding protons on addition of mild acids (CF_3_CH_2_OH)^11^ and further increases in activity were realised when these acidic groups were added to the surrounding ligand.^9^

One of the most well-studied transition metal catalysts is Ni(cyclam)^2+^ which demonstrates very good CO selectivity at relatively low overpotentials in aqueous conditions. Most studies have been conducted at a Hg electrode due to the large negative potential window. Furthermore, Ni(cyclam)^+^ has been shown to adsorb to the Hg electrode and increase its reactivity to CO_2_ as a result.^12^ Recent studies by Kubiak and co-workers have demonstrated effective CO_2_ reduction at a glassy carbon electrode^13^ with the catalyst efficiency boosted by a CO scavenger [Ni(tetramethylcyclam)]^2+^.^14^

Our interest was to apply the electrochemical reduction to carbon-11 CO_2_ ([^11^C]CO_2_) generating [^11^C]CO. The range of functionalities that can be synthesised from [^11^C]CO make it an attractive precursor for positron emission tomography (PET) radiotracer development.^15,16^ However, the poor solubility of [^11^C]CO in organic solvents and low partial pressure, adds to the challenge of a short half-life (*t*_1/2_ = 20.4 min). A number of methodologies have been developed to convert cyclotron-produced [^11^C]CO_2_ to [^11^C]CO: (1) gas phase reduction method, which involves passing [^11^C]CO_2_ through a heated column of zinc or molybdenum at 400 °C or 850 °C respectively.^17^ Whilst molybdenum is preferred, both methods suffer reliability and repeatability issues making clinical production difficult from a regulatory stand-point; (2) chemical reduction methods that have been trialled use reactive silane lithium reagents that must be prepared beforehand.^18^

The aim of this work was to conduct a proof-of-principle study into the viability of electrochemical [^11^C]CO_2_ reduction to [^11^C]CO within a radiochemical setting. Trapping efficiencies represent decay-corrected trapped radioactivity as a percentage of dispensed radioactivity. RCY's are decay corrected and are estimated from dispensed [^11^C]CO_2_ converted to [^11^C]*N*-benzylbenzamide (**5**).

A DropSens® screen printed electrode with a carbon working electrode (WE), a counter electrode (CE) and a silver reference electrode (RE) was used for the electrochemical conversion (Fig. 1A). Fig. 1B and C show how the electrodes and electrode connector fit inside Vial A.

**Fig. 1:**
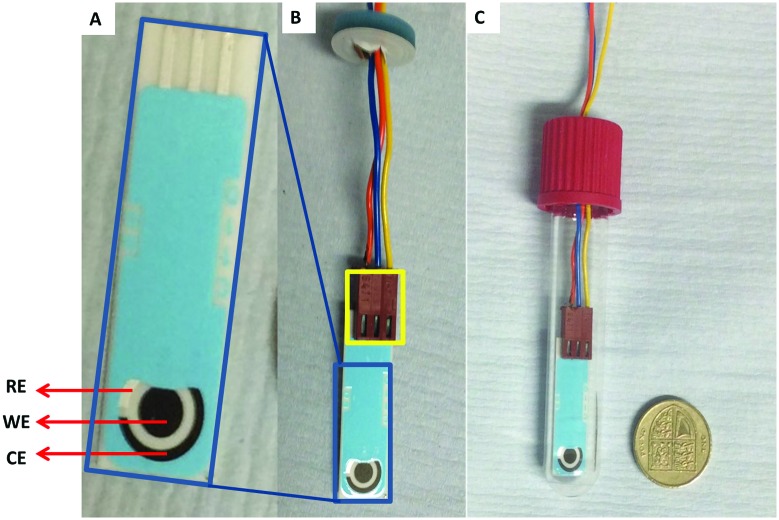
(A) DropSens® screen printed electrode with carbon working electrode (WE), a counter electrode (CE) and a silver reference electrode (RE). (B) Screen printed electrode attached to cables *via* electrode connector (yellow rectangle) to the potentiostat through a silicone septum. (C) Electrode and connectors inserted in custom made screw-top vial.

Initial experiments were conducted using a two vial set-up (set-up I – Vials A and B, Fig. 2). Vial A (used to convert [^11^C]CO_2_ to [^11^C]CO) contains the electrodes and the electrocatalysts Ni(cyclam)^2+^ or Zn(cyclen)^2+^ (**1–2**, Scheme 1) complexes in 0.1 M KCl_(aq.)_ solution at 20 °C.^19^ Vial B (used to trap and fix [^11^C]CO) containing the carbonylation reagents to produce [^11^C]*N*-benzylbenzamide ([^11^C]**5**, Scheme 1).^20^ An ascarite trap was placed between the two vials to capture any untrapped [^11^C]CO_2_ (Fig. 2).

**Fig. 2:**
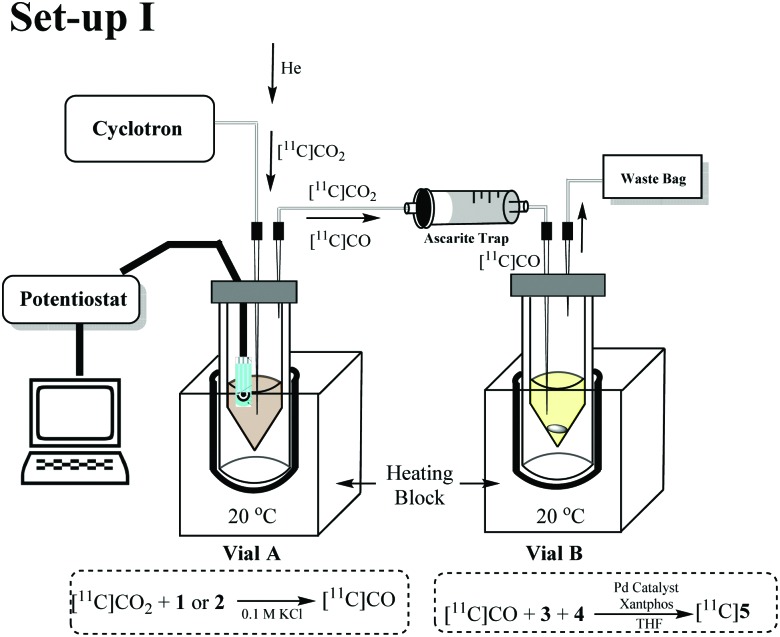
Schematic of the two-vial, one-valve setup. (1) [^11^C]CO_2_ delivered to Vial A with potentiostat switched on for 5 min (prior to and during delivery). (2) Helium sweep gas applied for 30 s through Vial B. (3) Carbonylation reaction in Vial B conducted for 10 min at 40 °C.

**Scheme 1:**
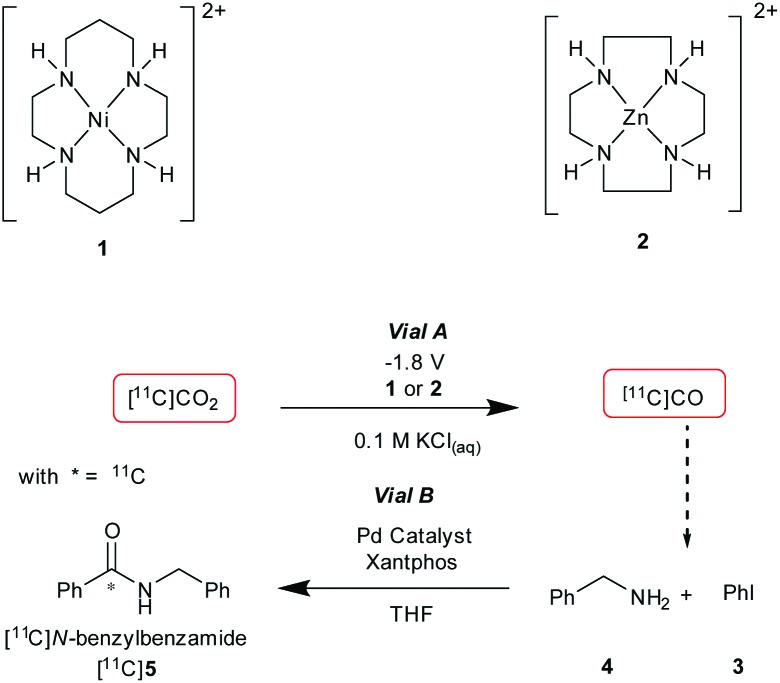
Vial A: catalyst **1** or **2**, 0.1 M KCl, –1.8 V for 5 min. Vial B: [^11^C]CO, iodobenzene (**3**, 0.01 mmol), benzylamine (**4**, 0.46 mmol), [(Cinnamyl)PdCl]_2_ (0.007 mmol), Xantphos (0.007 mmol), THF (0.5 mL), 20 °C, 6 min.

The setup tested is shown in Fig. 2. As the first experiment, [^11^C]CO_2_ was bubbled through the system with no potential applied to the electrodes. A low percentage (<1%) of [^11^C]**5** (Table 1, entry 1) was detected and this was believed to be from cyclotron generated [^11^C]CO. When a potential of –1.8 V *vs.* Ag/AgCl was applied (in non-radioactive experiments (see ESI†) potentials of –1.4 and –1.6 V were used to allow full quantification of CO production, at –1.8 V, the detector was quickly saturated by CO. At more negative potentials H_2_ production was thought to become more favoured), [^11^C]**5** was produced with high RCP's (>98%) but low RCY's (Table 1, entries 2 and 3). The low RCY was thought to be due to the low trapping efficiency of [^11^C]CO_2_ within Vial A. From these preliminary results it appeared that complex **2** performed marginally better than complex **1**. The predicted trapping of [^11^C]CO as an adduct of **1^14^** (ESI,† S2) was not observed in any usable quantity so experiments were conducted with **2**.

**Table 1:** Results of preliminary electrolysis experiments

Entry	Complex	*E*_app_ (V)	Radioactivity Vial A remaining at EOS (%)	Radioactivity Vial B at EOS (%)	RCP [^11^C]**5** ^*a*^ (%)	RCY [^11^C]**5** ^*b*^ (%)
1	**1**	0	5.5	0.5	75	<1
2	**1**	–1.8	3.5	7.2	97	7
3	**2**	–1.8	1.2	9.8	98	10

^*a*^Radiochemical purity (RCP) of [^11^C]**5** determined by analytical radio HPLC.

^*b*^Decay corrected radiochemical yields (RCY) are based on the radioactivity of Vial B multiplied by the radiochemical purity of [^11^C]**5** compared to the total radioactivity measured at end of cyclotron target bombardment (EOB). *n* = 2. End of synthesis (EOS).

In order to evaluate and improve the trapping of [^11^C]CO_2_ in Vial A we designed a two-vial, one-valve set-up (set-up II) shown in Fig. 3. During [^11^C]CO_2_ delivery, Vial A was connected to ascarite 1 (Eckert & Ziegler Modular-Lab). By placing ascarite 1 after Vial A, the amount of [^11^C]CO_2_ trapped in Vial A before starting the electrolysis step could be assessed. At end of delivery (EOD) electrolysis would begin at –1.8 V and on completion of electrolysis, the valve was moved to divert gases ([^11^C]CO_2_ and [^11^C]CO) to Vial B by Helium purge. Ascarite 2 was placed after Vial B so that relative amounts of [^11^C]CO_2_ and [^11^C]CO (the latter assumed to be converted to [^11^C]**5**) in Vial B could be established by radio HPLC (see ESI†).

**Fig. 3:**
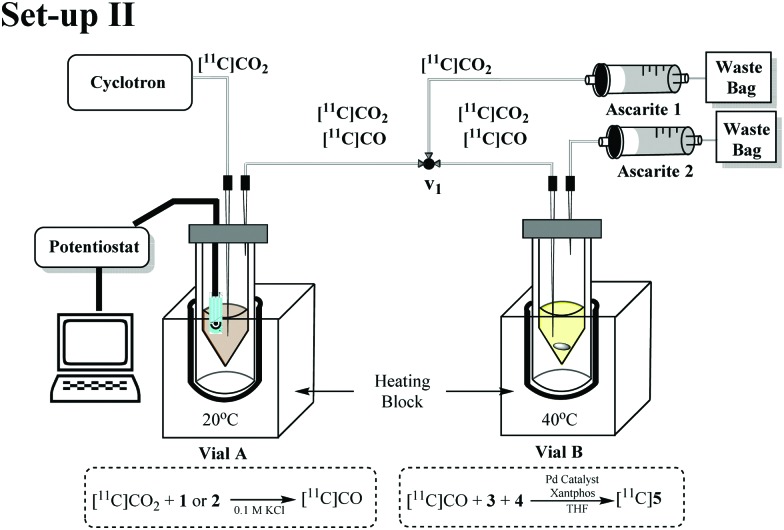
Schematic of the two-vial, one-valve setup. (1) [^11^C]CO_2_ delivered to Vial A. (2) potentiostat switched on for 10 min. (3) He sweep gas applied for 10 s through Vial B. (4) carbonylation reaction in Vial B conducted for 10 min at 40 °C.

The performance of set-up II was evaluated using complex **2** at 150 and 15 mM (Table 2, entries 1 and 2). Increasing the concentration of **2** from 15 to 150 mM resulted in higher trapping (56% and 66% respectively) and conversion (<1% and 4% RCY, respectively). The improvements in trapping [^11^C]CO_2_ have been previously achieved using bases such as diazabicyclo[5.4.0]undecene (DBU), 2-*tert*-butylimino-2-diethylamino-1,3-dimethylperhydro-1,3,2-diazaphosphorine (BEMP), tetramethylethylenediamine (TMEDA)^21^ and triethanolamine (TEA).^22^ For our study we chose the strong base DBU and a weaker base TEA.

**Table 2:** Conditions and results using Set-up II for the reduction of [^11^C]CO_2_ to [^11^C]CO by **2** and subsequent [^11^C]CO capture as [^11^C]**5**

Entry	**2** (mM)	Base (mM)	HCl added (0.1 N, 0.2 mL)	Est. [^11^C]CO_2_ trapping in Vial A at EOD^*a*^ (%)	RCP^*b*^ [^11^C]**5** (%)	RCY [^11^C]**5** ^*c*^ (%)
1	150	—	—	66	7	4
2	15	—	—	56	3	1
3	150	DBU (75)	—	80	—	—
4	150	DBU (75)	✓	48	3	3
5	150	DBU (7.5)	✓	18	—	—
6	150	TEA (75)	—	23	—	—
7	150	TEA (75)	✓	65	29	1
8	150	TEA (7.5)	✓	60	7	5
9	15	TEA (75)	✓	32	10	<1
10	15	TEA (7.5)	✓	35	8	2
11^*d*^	150	—	—	12	8	3
12^*d*^	150	TEA (75)	✓	64	5	<1
13^*d*^	15	TEA (7.5)	✓	52	20	6

^*a*^Trapping in A = % radioactivity in Vial A *versus* total radioactivity released by the cyclotron.

^*b*^RCP determined by analytical radio-HPLC.

^*c*^Radiochemical yield (RCY) = [(radioactivity in Vial B × RCP [^11^C]**5**)/(radioactivity in Vial A at EOD) × 100].

^*d*^Only WE and CE used.

The next experiments were performed using 150 mM of **2**. To improve [^11^C]CO_2_ trapping, DBU was added. A high concentration of base was used initially to improve the trapping of [^11^C]CO_2_ and subsequently promote the formation of [^11^C]**5**. Although 75 mM of bases increased the trapping of [^11^C]CO_2_ (Table 2, entry 1 *versus* entry 3) the high concentration appeared to prevent the formation of [^11^C]CO. No conversion was observed until HCl was added (0.2 mL of 0.1 N HCl, Table 2, entry 4). This was thought to be due to a change of pH from 6.5 to 12–13 from using DBU in water solution. The resultant acidification of Vial A (on addition of HCl) released the trapped [^11^C]CO_2_ so it was free to be converted to [^11^C]CO.

On addition of HCl, entry 4 showed a RCY of 3%. A lower concentration of base (7.5 mM) was used to investigate if the concentration of **2** and the concentration of base had an optimum combination, perhaps acting through the base binding with **2**. Substitution of DBU for TEA was added as a more moderate base (pH 9–10) and the trapping efficiency varying from 23–65% in Vial A (Table 2, entries 6–10). Table 2, entries 7 and 8, showed trapping efficiencies of ∼60%. On reducing the concentration of **2** to 15 mM, the trapping efficiency is halved (∼30%) irrespective of the concentration of TEA suggesting that the concentration of **2** plays a larger role than TEA in trapping [^11^C]CO_2_. This variety of trapping efficiencies shown in Table 2 was thought to be a consequence of the high flow rate (50 ml min^–1^) of [^11^C]CO_2_ into an aqueous solution.^23^

The optimum RCY achieved (Set-up II, Fig. 3) was 5% (Table 2, entry 8) which was obtained when 7.5 mM of TEA was used. These results appeared to show that a compromise of a milder base would still facilitate reasonable trapping whilst not hindering [^11^C]CO_2_ reduction. In order to simplify the reaction set-up and increase electrode surface area, a 2-electrode set-up was used in Vial A. This involved using just the working electrode (WE) and the counter electrode (CE) (Table 2, entries 11–13). The optimum conversion achieved by the 2-electrode cell was 6% (Table 2, entry 13) with 52% of [^11^C]CO_2_ initially trapped in Vial A.

In conclusion, the first electrochemical [^11^C]CO_2_ to [^11^C]CO reduction has been achieved with a 2-vial set-up to incorporate the [^11^C]CO product into [^11^C]*N*-benzylbenzamide in a proof-of-principle study. **2** showed good [^11^C]CO_2_ trapping and conversion to [^11^C]CO. The effectiveness of **2** compared to **1** for [^11^C]CO_2_ reduction was surprising and further studies are needed to investigate this fully although we believe that ZnO nanoparticles are being generated at the electrode. Improvements in the performance of **1** could come from binding the catalyst to the electrode.^8,25^ Furthermore, the application of a two-electrode design of Vial A was shown to be viable. We believe that a pre-concentration step of [^11^C]CO_2_ prior to vial A would lead to better performance both in trapping of [^11^C]CO_2_ and conversion.

This work was supported by the Medical Research Council through financial support (MRC-1527506 and MR/K022733/1) and the Institute of Chemical Biology at Imperial College London. The authors acknowledge financial support from the Department of Health *via* the National Institute for Health Research (NIHR) comprehensive Biomedical Research Centre award to Guy's & St Thomas' NHS Foundation Trust in partnership with King's College London and King's College Hospital NHS Foundation Trust and the Centre of Excellence in Medical Engineering funded by the Wellcome Trust and EPSRC under grant number WT 088641/Z/09/Z.
