# Supplementary material for: Electrochemical [11C]CO2 to [11C]CO conversion for PET imaging
Source: Chem Commun (Camb). 2017 Feb 24;(20):2982–5. doi: 10.1039/c7cc00319f (PMC5633808; doi:10.1039/c7cc00319f)
Supplement: Supplementary file 1 [file CC-053-C7CC00319F-s001.pdf]

Electronic Supplementary Material (ESI) for ChemComm.
This journal is © The Royal Society of Chemistry 2017

Supplementary Material (ESI)

Electrochemical [^{11}C]CO_2 to [^{11}C]CO conversion for PET imaging

Supporting Information

David A. Anders,[a,b] Salvatore Bongarzone,[b] Robin Fortt,[b] Antony D. Gee[b,*] and Nicholas J. Long[a,*]

a. Department of Chemistry, Imperial College London, London SW7 2AZ, UK (Email: n.long@imperial.ac.uk

b. Division of Imaging Sciences and Biomedical Engineering, King's College London, 4th Floor Lambeth Wing, London SE1 7EH, UK

Contents

General method and materials....1

Synthesis of reduction catalysts **1** and **2** 2

Synthesis of **1**....2

Synthesis of **2**....2

Non-radioactive controlled potential electrolysis experiments on **1** and **2** 3

Non-radioactive test of the DropSens C110 screen printed electrode....6

Radiochemistry vial A preparation 6

Radiochemistry vial B preparation 6

Synthesis of [^{11}C]**5** by two vial method....7

Notes and References....8

General method and materials

All chemicals and dry solvents were purchased from Sigma-Aldrich and Alfa Aesar and used as received. HPLC analysis was performed on an Agilent 1200 system equipped with a UV detector (λ=254 nm) and a β+-flow detector coupled in series. A reverse-phase column (Agilent Eclipse XDB-C18, 4.6 x 150 mm, 5 µm) was used with a flow rate of 1 mL min^{-1}. The gradient for **5** was linear between 10–90% over 5 min (CH_3CN:0.1 M NH_4HCO_2, 10:90), isocratic in between 5–9 min (CH_3CN:0.1 M NH_4HCO_2, 90:10) and linear in between 9–13 min (CH_3CN:0.1 M NH_4HCO_2, 90:10). Identification of all radioactive products was confirmed by co-elution with the corresponding non-radioactive compounds. The reference compound **5** elutes at RT = 5.6 min. ^{1}H NMR spectra were obtained using a BRUKER AVANCE

AV 400 MHz spectrometer. Mass spectrometry was performed using on a Water LCT premier ES-TOF. Controlled-potential electrolysis (CPE) was carried out in a three-necked flask with a three-electrode set-up using carbon paper as working electrode and Pt grid counter electrode with a Ag/AgCl/KCl(sat) reference electrode. The Faradaic yield was calculated from the amount of CO or H_2 accumulated in the headspace (60 mL), as measured by GC (Perkin-Elmer Clarus 580) fitted with a dual-ionisation detector (DID) connected to (molecular sieve column) to detect H_2, CO, O_2, CH_4 and N_2 and flame ionization detector (FID) connected to 1/8" SF x 5' Unibeads column to detect hydrocarbons. Helium was used as the GC carrier gas. Ignacio Garcia at Imperial recorded all XP spectra using a K-alpha$^+$ XPS spectrometer equipped with a MXR3 Al Kα monochromated X-ray source (hν = 1486.6 eV). X-ray gun power was set to 72 W (6 mA and 12 kV). With this X-ray settings, the intensity of the Ag $3d_{5/2}$ photoemission peak for an atomically clean Ag sample, recorded at 20 eV pass energy (PE), was 5×10^6 counts s^{-1} and the full width at half maximum (FWHM) was 0.58 eV. Binding energy calibration was made using Au $4f_{7/2}$ (84.01 eV), Ag $3d_{5/2}$ (368.20 eV) and Cu $2p_{3/2}$ (932.55 eV). The pressure during the measurement of XP spectra was $\leq 1 \times 10^{-8}$ mbar. All XP spectra were charge corrected by referencing the fitted contribution of C-C graphitic-like carbon in the C 1s signal to 285 eV. $[^{11}C]CO_2$ was produced using a Siemens RDS112 cyclotron by the 11 MeV proton bombardment of nitrogen (+ 1% O_2) gas via the $^{14}N(p,\alpha)^{11}C$ reaction in an aluminium target with HAVAR front foil which was helium cooled on the front face. All source gasses were 9.9999% purity (BOC N6) and were used as received. The cyclotron-produced $[^{11}C]CO_2$ was bubbled in a stream of helium gas with a flow rate of 50 mLmin^{-1} post target depressurisation. The molar radioactivity of $[^{11}C]$**5** (for entry 2 in Table 2) was estimated to be 56 GBq μmol^{-1} (n=1).

Synthesis of reduction catalysts 1 and 2

Synthesis of 1[1]

Nickel(II) chloride hexahydrate (0.29 g, 2.2 mmol, 1.8 eq.) was dissolved in warm ethanol (20 mL) and was added to a solution of cyclam (0.25 g, 1.2 mmol, 1 eq.) in ethanol (10 mL). The resulting light brown solution was stirred at 40 °C for 20 min. Diethyl ether (30 mL) was added to precipitate the product which was filtered and washed with diethyl ether (3 x 10 mL). This product was recrystallized by adding ether to a saturated solution of the product in methanol to yield pure product as a lilac solid (0.27 g, 0.82 mmol, 68% yield); ESI-HRMS: $[M+MeCN]^{2+}$ = m/z 149.5263). Anal. Calcd. For $C_{10}Cl_2H_{24}N_4Ni$: C, 36.40; H, 7.33; N, 16.98. Found: C, 36.46; H, 7.25; N, 16.90.

Synthesis of 2[2]

Cyclen (1.0 g, 5.8 mmol, 1 eq.) was dissolved in ethanol (10 mL) in a scintillation vial equipped with a stirrer bar. The colourless solution was heated and stirred at 65 °C using an oil bath. $Zn(ClO_4)_2 \cdot 6H_2O$ (2.6 g, 7.0 mmol , 1.2 eq.) was dissolved in ethanol (3 mL), and this colourless solution was slowly added to the ligand at 65 °C dropwise. Upon zinc addition, the colourless mixture became turbid and a white precipitate formed. The suspension was stirred at 65 °C for 2 h. The clear solution was left overnight to crystallise at ambient temperature. The white solid was filtered and washed with cold ethanol (3 × 30 mL). The resulting white solid was dried under vacuum ambient temperature to yield the final product as a white, granular solid Yield: 1.81 g (4.2 mmol, 84%). ^1H NMR (400 MHz, D_2O,

major conformer) δ 2.97−2.93 (m, 8 H), 2.85−2.79 (m, 8 H); ESI-HRMS: calcd for $C_{12}H_{26}ZnN_6$ = 159.0755, found *m/z* = 159.0747 [Zn(cyclen)$^{2+}$ + $2CH_3CN$], Anal. Calcd. for $C_8Cl_2H_{20}N_4O_8Zn$: C, 22.01; H, 4.62; N, 12.83. Found: C, 21.86; H, 4.75; N, 12.75.

Non-radioactive controlled potential electrolysis experiments on 1 and 2

Controlled-potential electrolysis (CPE) was carried out in a three-necked flask with a three-electrode set-up using carbon paper as working electrode and Pt grid counter electrode with a Ag/AgCl/KCl(sat) reference electrode. The Faradaic yield was calculated from the amount of CO or H_2 accumulated in the headspace (60 mL), as measured by GC (Perkin-Elmer Clarus 580) fitted with a dual-ionisation detector (DID) connected to (molecular sieve column) to detect H_2, CO, O_2, CH_4 and N_2 and flame ionization detector (FID) connected to 1/8" SF x 5' Unibeads column to detect hydrocarbons. Helium was used as the GC carrier gas.

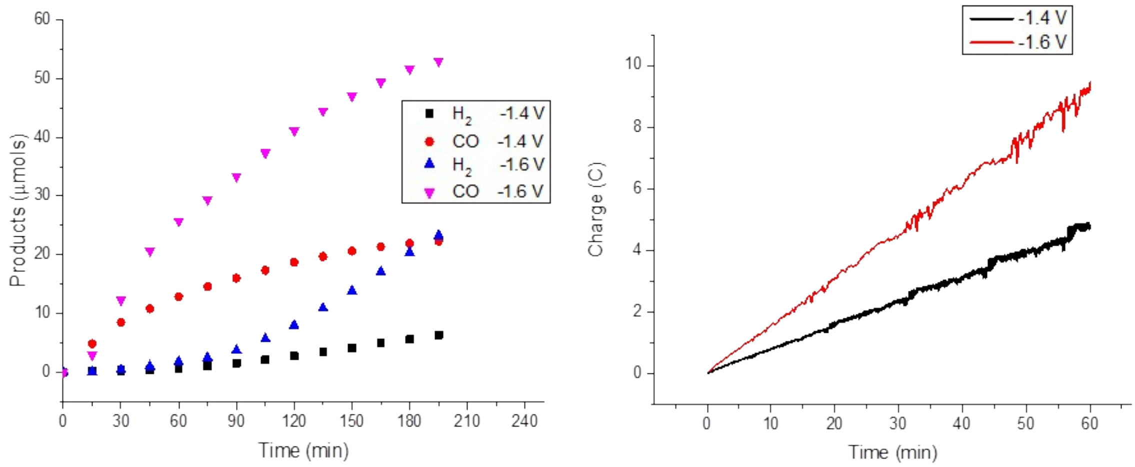

Figure S1: (Left) Graph of H_2 and CO production in controlled potential electrolysis experiments on 1 (25 ml, 5 mM) at -1.4 V and -1.6 V. (Right) Cumulative charge passed during controlled potential electrolysis. Carbon paper working electrode, Ag/AgCl reference electrode and Pt grid counter electrode were used in this experiment. Flow rate of CO_2 1 ml min^{-1}

In non-radioactive electrolysis experiments with both catalyst **1** and **2** at -1.8 V the GC detector became saturated and so -1.4 and -1.6 V were used. In radiochemical experiments it was decided that -1.8 V would give better [^{11}C]CO production.

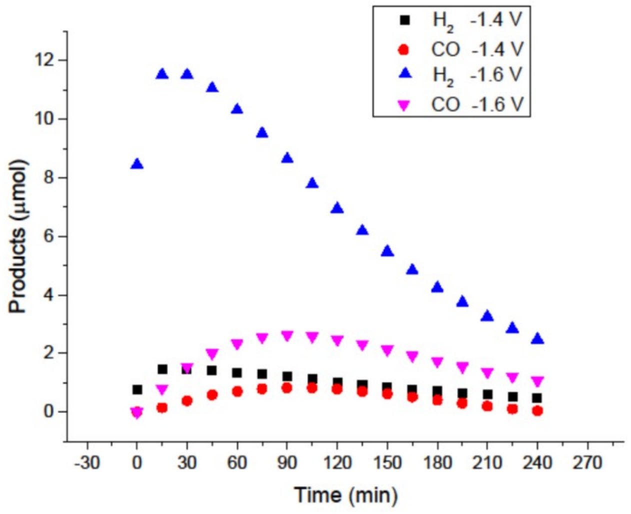

Figure S2: Gas chromatography results of the CO release. After electrolysis shown in S1 the potentiostat remained at the potential shown whilst the electrolysis cell was purged for 45 minutes by N_2. Time = 0 when potentiostat switched off and purging N_2 flow rate brought back to 1 ml min^{-1}

Figure S2 appears to show degradation of Ni(cyclam)(CO) adduct.

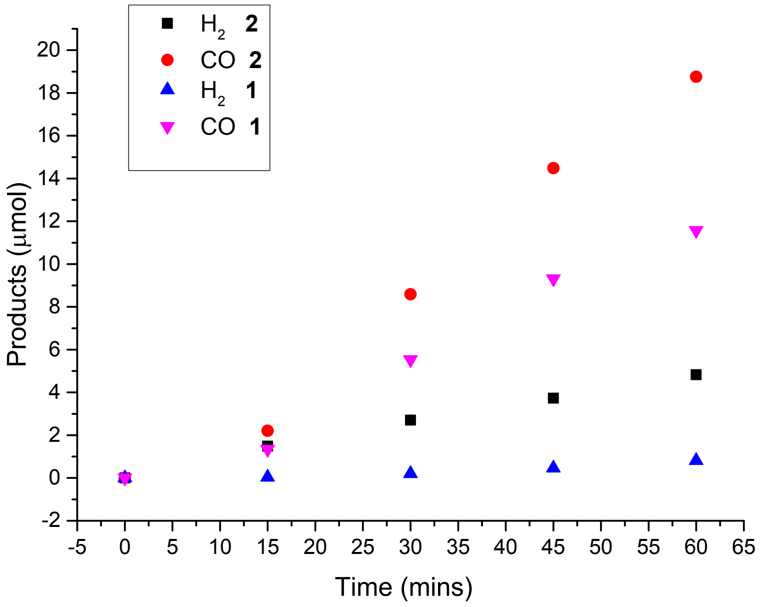

Figure S3: Graph to compare initial rates of electrolysis experiments for 1 and 2 (25 ml, 5 mM). All experiments run at -1.6 V in 0.1 M KCl. Carbon paper working electrode, Ag/AgCl reference electrode and Pt grid counter electrode were used in this experiment

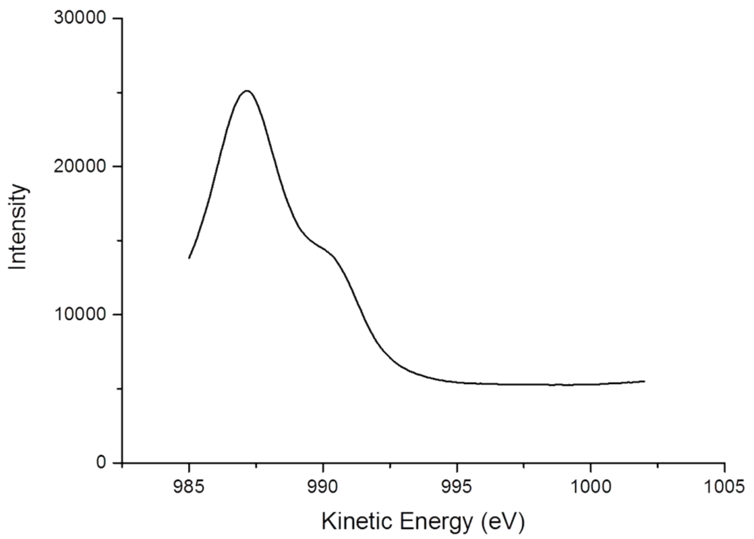

Figure S4: XPS spectrum of working electrode in the LMM region after electrolysis with 2 (50 mM)

Figure S4 shows that the characteristic band of ZnO can be clearly seen with a peak at 988 eV and shoulder at 990 eV.

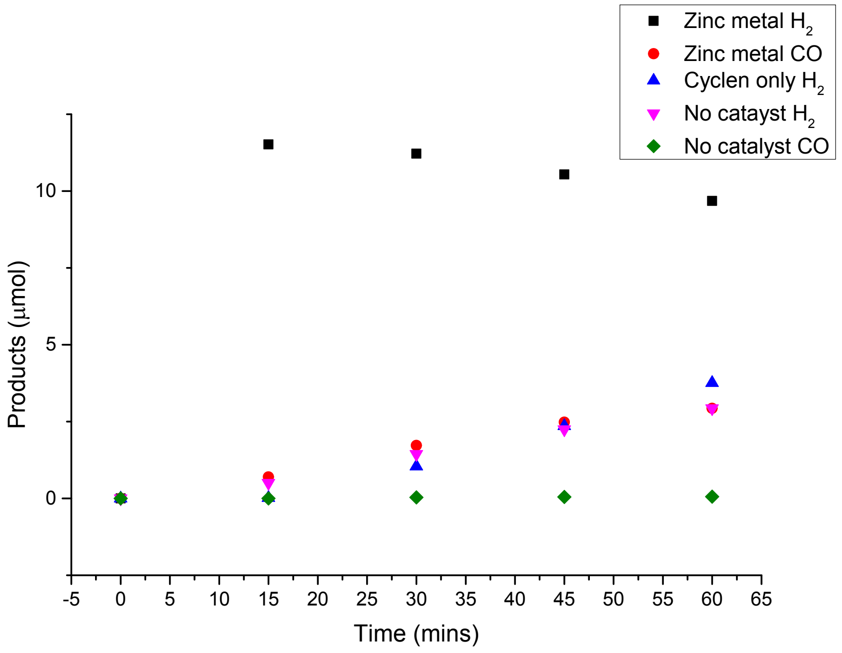

Figure S5: Controlled potential electrolyses run at -1.6 V in 0.1 M KCl for 1 hour as control experiments. Carbon paper working electrode, Ag/AgCl reference electrode and Pt grid counter electrode were used in this experiment

Non-radioactive test of the DropSens C110 screen printed electrode

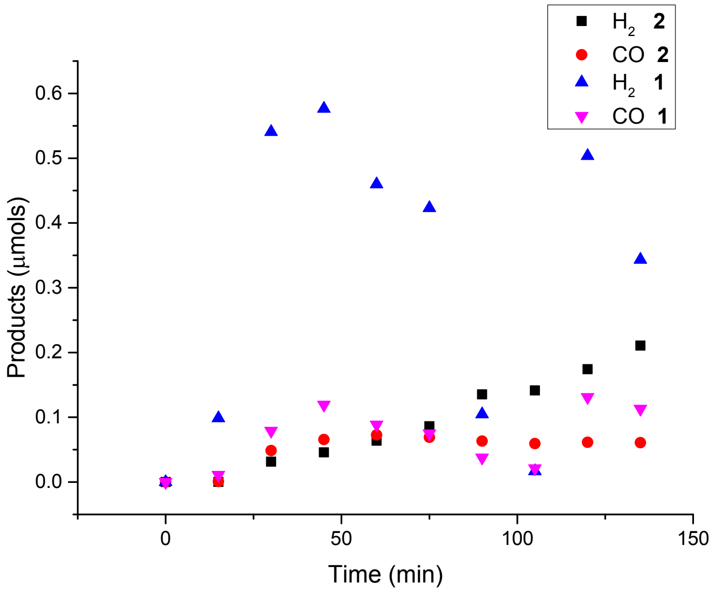

Figure S6: Controlled potential electrolyses run at -1.6 V in 0.1 M KCl under CO_2 with 1 (1 ml, 5 mM) or 2 (1 mL, 5 mM) using DropSens screen printed carbon working and counter electrode with a silver reference electrode

Radiochemistry vial A preparation

The screen printed electrodes (DRP-C110) were provided by DropSens. The electrode connector was made in-house. This was done by taking a 3-way 1-row housing (yellow box) (RS components, Stock No. 360-6061). The 3-way housing had connector pins in line with the 3 connectors on the electrode and a good electrical connection was maintained by cutting a 0.5 mm slit in the connector (same width as the electrode). Each connector pin was individually connected to the potentiostat (Ivium CompactStat). The 3-way connector sits at the top of the reaction vessels and the wires pass through the silicone washer to maintain an airtight seal. The electrolysis vial (Fig 1.A-C) was custom made with a screw top (GL18 thread, 16 mm OD, 12.4 mm I.D, 100 mm long) (GPE Scientific Ltd, Leighton Buzzard, UK). All the lines used for gas transfer were PTFE tubing (length: 10–20 cm, O.D.: 0.79 x 0.4 in., I.D.: 1/32 x 0.16 in.). The exception was for the electrolysis entry line, PEEK tubing (length: 10–20 cm, O.D.: 0.79 x 0.4 in., I.D.: 1/32 x 0.16 in) was used and this was also fitted with a one-way flow valve.

Radiochemistry vial B preparation

Carbonylation vial was charged with iodobenzene (**3**) (1.12 µL, 0.01 mmol, 1 eq.), benzylamine (**4**) (50.24 µL, 0.46 mmol, 46 eq.), [(cynnamyl)PdCl]$_2$ (3.6 mg, 0.007 mmol, 0.07 eq.), xantphos (4.0 mg, 0.007 mmol, 0.07 eq.) in dry THF (0.5 mL) under argon atmosphere.[3]

Crimp caps (centre hole with 3.0 mm PTFE seal aluminium silver 20 mm, Fisherbrand, 10132712) and oven dried vials (KX Microwave Vials, 10 mL) were used for the carbonylation vial. The electrolysis vial and the carbonylation vial were placed in two heating blocks of a Eckert and Ziegler Modular-Lab equipped with magnetic stirring

Synthesis of [^{11}C]5 by two vial method

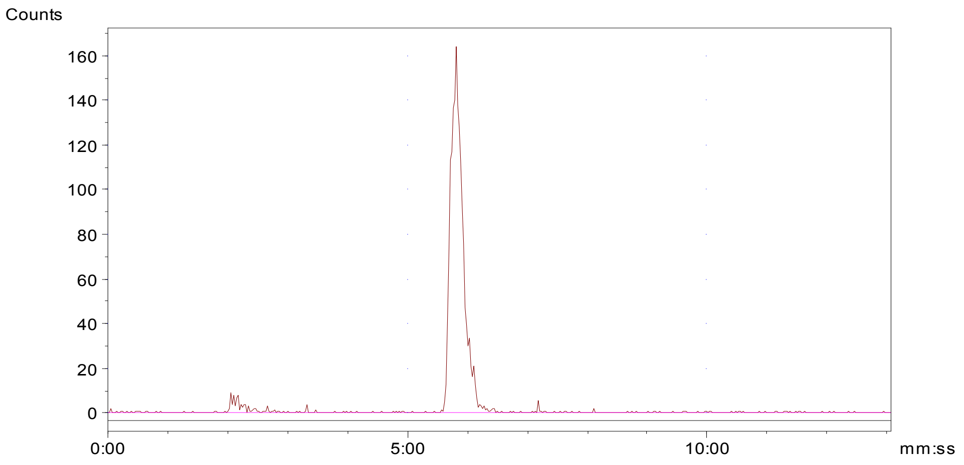

Figure S7: Radio-HPLC of the [^{11}C]5 using Set-up I (Table 1, entry 3)

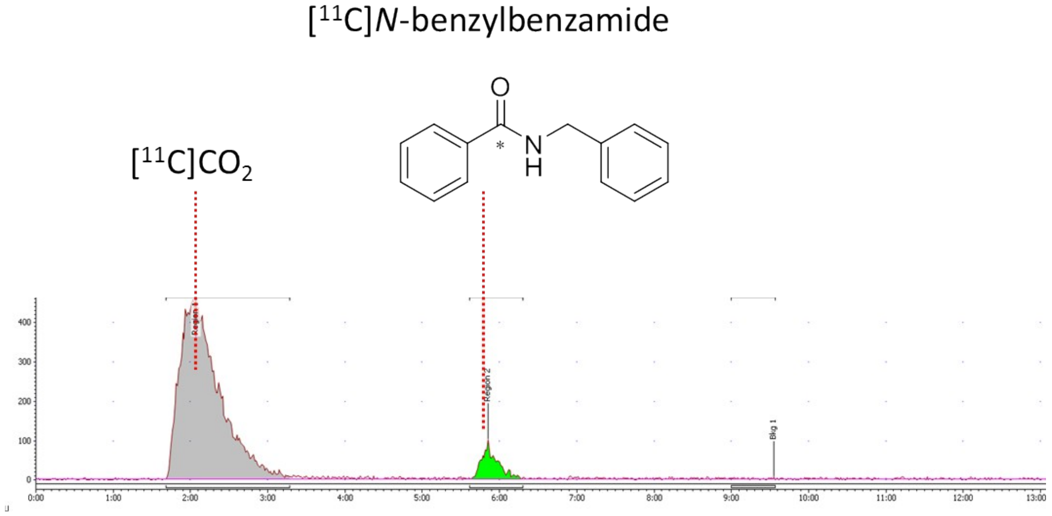

Figure S8: Radio-HPLC of the [^{11}C]5 using Set-up II (Table 2, entry 9)

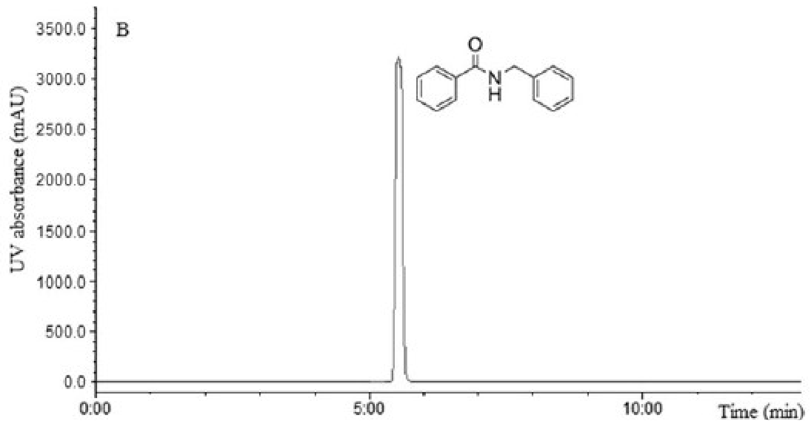

Figure S9: HPLC of reference compound 5

Notes and References

1. J. L. Karn and D. H. Busch, *Inorg. Chem.*, 1969, **8**, 1149–1153.

2. L. Koziol, C. A. Valdez, S. E. Baker, E. Y. Lau, W. C. Floyd, S. E. Wong, J. H. Satcher, F. C. Lightstone and R. D. Aines, *Inorg. Chem.*, 2012, **51**, 6803–6812.

3. C. Taddei, S. Bongarzone, A. K. Haji Dheere and A. D. Gee, *Chem. Commun.*, 2015, **51**, 11795–11797.

4. The delay between UV detector and radio-detector at flow rate on 1 mLmin^{-1} is 0.5 min.
